# Identification of Telomerase RNAs from Filamentous Fungi Reveals Conservation with Vertebrates and Yeasts

**DOI:** 10.1371/journal.pone.0058661

**Published:** 2013-03-14

**Authors:** Paulius V. Kuprys, Shaun M. Davis, Tyler M. Hauer, Max Meltser, Yehuda Tzfati, Karen E. Kirk

**Affiliations:** 1 Department of Biology, Lake Forest College, Lake Forest, Illinois, United States of America; 2 Department of Genetics, The Silberman Institute of Life Sciences, The Hebrew University of Jerusalem, Givat Ram, Jerusalem, Israel; Tulane University Health Sciences Center, United States of America

## Abstract

Telomeres are the nucleoprotein complexes at eukaryotic chromosomal ends. Telomeric DNA is synthesized by the ribonucleoprotein telomerase, which comprises a telomerase reverse transcriptase (TERT) and a telomerase RNA (TER). TER contains a template for telomeric DNA synthesis. Filamentous fungi possess extremely short and tightly regulated telomeres. Although TERT is well conserved between most organisms, TER is highly divergent and thus difficult to identify. In order to identify the TER sequence, we used the unusually long telomeric repeat sequence of Aspergillus oryzae together with reverse-transcription-PCR and identified a transcribed sequence that contains the potential template within a region predicted to be single stranded. We report the discovery of TERs from twelve other related filamentous fungi using comparative genomic analysis. These TERs exhibited strong conservation with the vertebrate template sequence, and two of these potentially use the identical template as humans. We demonstrate the existence of important processing elements required for the maturation of yeast TERs such as an Sm site, a 5′ splice site and a branch point, within the newly identified TER sequences. RNA folding programs applied to the TER sequences show the presence of secondary structures necessary for telomerase activity, such as a yeast-like template boundary, pseudoknot, and a vertebrate-like three-way junction. These telomerase RNAs identified from filamentous fungi display conserved structural elements from both yeast and vertebrate TERs. These findings not only provide insights into the structure and evolution of a complex RNA but also provide molecular tools to further study telomere dynamics in filamentous fungi.

## Introduction

Telomeres are protective structures at the ends of linear chromosomes [1], [2]. The telomeric DNA consists of a tandemly repeated sequence, which varies in nucleotide composition and length depending on the organism. As cells replicate their DNA during each cell division cycle, their telomeres progressively shorten [3]. To mitigate telomere sequence loss, a specialized ribonucleoprotein, telomerase, lengthens telomeric DNA at the 3′ ends, thereby preventing any detrimental effects on genome stability triggered by excessive telomere shortening [4], [5].

The telomerase catalytic core enzyme is composed of the telomerase reverse transcriptase (TERT), and the telomerase RNA (TER). TER contains a template sequence for telomeric repeat synthesis catalyzed by TERT during telomere elongation. TERT proteins have been identified in genomes ranging from fungi to humans with conserved reverse-transcriptase and telomerase-specific protein motifs [6]. However, TERs have been particularly difficult to identify since there is poor sequence conservation between organisms. The first TER was discovered in the ciliated protozoan, Tetrahymena thermophila [7]. Subsequently, TERs have been identified from other ciliates [8], [9], [10], [11], [12], [13], vertebrates [14], yeasts [15], [16], [17], [18], [19], plants [20], and very recently in filamentous fungi [21]. Since some filamentous fungi, such as the Aspergilli, possess extremely short and tightly regulated telomeres [22], [23], they provide a unique model system to study telomere dynamics.

The telomerase RNAs between diverse organisms differ markedly not only in primary sequence but also in length, ranging from ∼150 nucleotides in *Tetrahymena thermophila* [7] to ∼2030 nucleotides in *Candida glabrata* [24]. However, all TERs discovered to date exhibit similar secondary structure motifs that are involved in various aspects of TER function. First, they contain a template that is used to synthesize new telomeric repeats. The template region includes more than one complete telomeric repeat but no more than two. The 3′ end of the template repeats the sequence at the 5′ end, within a single-stranded region which allows for initial binding and alignment to the overhanging telomere, followed by a subsequent translocation event [25]. Second, TERs possess a template boundary, which has been identified or predicted in yeasts, ciliates, and vertebrate TERs [26], [27], [28]. The template boundary is located 5′ of the template and functions to prevent the excess copying of nucleotides beyond the template [26]. Third, a pseudoknot containing U-A·U base-triples has been identified in vertebrates, yeasts and ciliates [29], [30], [31]. The pseudoknot has been implicated in the proper orientation of the aligned template and telomeric 3′ end to the active site of TERT [32].

Further analysis of vertebrate TERs has established the presence of a stem-loop, p6.1 [33], while yeasts display an analogous stem, S3, that conserves several nucleotides with vertebrates within a similar arrangement of a three-way junction (TWJ) [16], [34]. These RNA structures are necessary for the assembly of TERT with TER and for the activation of telomerase [33]. In addition, despite no clear sequence or structure conservation, stem-loop IV in ciliates was predicted to serve similar functions [35]. Therefore, stem-loop IV, p6.1, and TWJ were suggested to be functional homologs and termed stem-terminus elements or assembly/activation stem-loops [36], [37].

In this study we report TER sequences from 13 different Aspergilli, which were sufficiently similar in sequence to facilitate phylogenetic covariation-based secondary structure analysis. Surprisingly, given the taxonomic diversity between the filamentous fungi and vertebrates, several features resembling vertebrate TERs, such as the template and the three-way junction, were found in the filamentous fungi TERs. In contrast, other features are more similar to yeast TERs, such as the overall length of the RNA, template boundary, Sm site and 5′ splice site, and branch point. These findings from the Aspergilli not only provide insights into the structure and evolution of a complex RNA molecule and its role in telomerase ribonucleoprotein function, but also provide a molecular tool in order to further study telomere dynamics in filamentous fungi such as *Aspergillus nidulans*.

## Materials and Methods

### Organism and Growth Media

Aspergillus oryzae RIB40 (ATCC) was grown on solid polypeptone dextrin (PD) media (polypeptone peptone 1%, dextrin 2%, KH_2_PO_4_ 0.5%, MgSO_4_ 0.05% casein hydrolysate 0.1%, NaNO_3_ 0.1%, agar 2%) modified from [38] and incubated at 30°C. After a week of growth, a spore stock was made. 0.2% tween 20 (polyoxyethylene-sorbitan monolaurate, Sigma) solution was applied to the plate, and rubbed with the flat edge of a Pasteur pipette. The spore stock mixture was washed three times in H_2_O and centrifuged for 3 minutes at 4500×g. A hemocytometer was used to determine the total concentration of the spore stock.

### RNA and DNA Isolation

5.2×10^5^ conidia from the spore stock were added to 50 mL of liquid PD media and incubated at 30°C with shaking (200 rpm) for 18–20 hours. The conidia were harvested by vacuum filtration over Miracloth (Calbiochem) and rinsed with water. Approximately 500 mg of the conidial growth mat was placed in a Lysing Matrix C tube (MP Biomedicals) and then placed in liquid nitrogen for one minute. 500 µL of RLC buffer with 2-mercaptoethanol (Qiagen) was added to the tube and placed into a Precellys 24 Tissue Homogenizer at 6000 rpm for 30 seconds. The homogenate was then used for RNA isolation using the RNeasy Plant Mini Kit (Qiagen). Isolated RNA was subsequently treated with Ambion DNA-free to remove any contaminating DNA and then stored at −80°C. Aspergillus oryzae RIB40 DNA was isolated using GeneClean (MP Biomedicals) and stored at −20°C. Nucleic acids were quantified by the BioSpec-nano spectrophotometer (Shimadzu).

### Querying for Potential *A. oryzae* TER Template Sequences

The genome of *A. oryzae* RIB40 was examined for two of its telomeric repeats (5′-TTAGGGTCAACATTAGGGTCAACA-3′) using the BLAST function from the National Institute of Technology and Evaluation (NITE). (http://www.bio.nite.go.jp/dogan/MicroTop?GENOME_ID=ao). Nineteen sequences were identified that contained a possible template. Sequences overlapping with a coding region were filtered out as well as sequences that were not conserved with A. flavus NRRL 3357, a very closely related taxonomic neighbor.

### Reverse Transcription

Primers (Operon Biotechnologies Inc.) were designed around the putative template region. Approximately 12–24 µg of RNA were added to NEBuffer 3 (New England BioLabs) and 40 U of RNase inhibitor (Ambion). As a control, RNase inhibitor was replaced with 50 U of RNase (New England BioLabs). Reactions were incubated for 15 minutes at 37°C and 5 minutes at 69.2°C in a Peltier Thermal Cycler (MJ Research). 3–6 µg of RNA from the previous reaction was incubated at 70°C for 4 minutes in the presence of 10 mM dNTPs (New England BioLabs), 2.5 µM forward primer (Table S1). Buffer, 40 U of RNase inhibitor (Ambion) and 200 U of M-MuLV Reverse Transcriptase (New England BioLabs) was added and incubated at 42°C for one hour, followed by incubation at 90°C for 10 minutes to inactivate the enzymes.

### PCR Conditions

Each tube contained 1 µL of cDNA (either RNased cDNA from reverse transcription, or cDNA from reverse transcription) or 150–300 ng of isolated *A. oryzae* DNA, in addition to 1.25 µM forward primer, 1.25 µM reverse primer (Table S1), 17 µl of nuclease free water, and 20 µl JumpStart REDTaq ReadyMix PCR Reaction Mix (Sigma). Nuclease-free water replaced DNA for the negative control. The following PCR cycle was used: 94°C for 5 minutes; 35 cycles at 94°C for 30 seconds, at 62°C for 30 seconds, and 72°C for 2 minutes; 72°C for 5 minutes.

### Rapid Amplification of cDNA Ends (RACE)

Approximately 10 µg and 1 µg of DNA-free RNA was used to complete 5′ RLM-RACE and 3′ RACE, respectively, following the protocol from the FirstChoice® RLM-RACE Kit (Ambion/Applied Biosystems) with primers found in Table S1.

### Poly (A) Tailing

The poly(A)-tailing protocol used was adapted from [21]. Approximately 20–40 µg of total *A. oryzae* RIB40 RNA was added to E. coli-PAP buffer, 10 mM ATP, 40 U of RNase inhibitor, and 5 U of E. coli-PAP enzyme. The tube was incubated at 37°C for 15 minutes. Subsequently polyadenylated RNA was used for the 3′ RACE reaction.

### Cloning

The inner 5′ RLM-RACE product and the outer 3′ RACE product were purified using GeneClean (MP Biomedicals). These products were cloned using TOPO TA Cloning kit (Invitrogen) transformed into OneShot chemically competent E. coli cells, spread onto LB plates containing kanamycin and incubated overnight at 37°C. Plasmid DNAs from transformed colonies were identified and isolated using the QIAprep Spin Miniprep Kit (Qiagen). DNA sequencing of cloned products was completed at the University of Chicago Cancer Research Center DNA Sequencing Facility.

### Querying of Genome Databases for TER Sequences

Using the entire DNA sequence region located between the C4-type Zn-finger protein and the TATA-binding interacting protein for *A. oryzae* RIB40 [39], sequenced genome databases were searched using the Basic Local Alignment Tool (BLAST). The search parameters used an expect value of 10 and a word size of 11. The following genome sequences were obtained from NCBI (http://www.ncbi.nlm.nih.gov): A. fumigatus Af293 [40], N. fischeri NRRL 181 [41], A. clavatus NRRL 1 [41], A. flavus NRRL3357, A. sojae NBRC 4239 [42], A. niger CBS 513.88 [43], A. kawachii IFO 4308 [44], Penicillium chrysogenum Wisconsin 54–1255 [45], A. nidulans FGSC A4 [46], [47]. The A. terreus NIH2624 sequence was obtained from the Aspergillus Genome Database (http://www.Aspergillusgenome.org), while the A. carbonarius ITEM 5010 v3 and A. aculeatus ATCC16872 v1.1 sequences were obtained from the DOE Joint Genome Institute (www.jgi.doe.gov).

### Sequence Analysis

Sequence alignments of the TERs were performed using ClustalX [48]. The alignments were used for common secondary structure prediction by RNAalifold [49]. Finally, based on the RNAalifold predictions, constrains were specified for MFold [50] to predict the secondary structures of conserved elements. Pairwise global alignment was completed using the Stretcher program from EMBOSS (http://www.ebi.ac.uk/Tools/psa) on the total estimated lengths of the *Aspergillus* TERs in comparison to the *A. oryzae* TER. The 5′ ends of TERs were defined by the distance from the conserved potential promoter 5′-CRCGDCGCG-3′ (where R is a purine and D is A, G, or T) 39–48 nt upstream of the 5′ end of the *A. oryzae* TER. The 3′ end of TERs were defined as the end of the Sm site.

### Sequence Deposition

The DNA sequences of the TERs have been deposited at GenBank (National Center for Biotechnology Information) under the following accession numbers: BK008581 (*A. oryzae* RIB 40), BK008635 (*A. fumigatus* Af293), BK008636 (*N. fischeri* NRRL 181), BK008637 (*A. clavatus* NRRL 1), BK008638 (*A. flavus* NRRL3357), BK008639 (*A.sojae* NBRC 4239), BK008640 (*A. niger* CBS 513.88), BK008641 (*A. kawachii* IFO 4308), BK008642 (*Penicillium chrysogenum* Wisconsin 54–1255), BK008643 (*A. nidulans* FGSC A4), and BK008644 (*A. terreus* NIH2624).

## Results and Discussion

### Identification of *A. oryzae* Telomerase RNA

All telomerase RNAs (TERs) discovered contain a template sequence, the function of which is to properly align the 5′ end of the template to the 3′ overhang of the telomere, allowing for multiple repeat synthesis [12], [14], [51], [52], [53], [54], [55]. To date all TERs contain more than one telomeric repeat in their template, so that at least 2 of the same nucleotides are repeated at the beginning and the end of the template [25]. We used the fortuitously long telomeric repeat of *Aspergillus oryzae*, 5′-TTAGGGTCAACA-3′, to first identify transcribed telomerase RNA candidates. Using two repeats of the *A. oryzae* telomeric sequence, the *A. oryzae* genome was searched using the Basic Local Alignment Search Tool (BLAST). Since the beginning and the end of the template region were unknown, the use of two repeats allowed for the identification of any permutation within these two repeats. This search yielded 19 possible TER template sequences that were further examined. The sequences were required to be present within an intergenic region. Finally, it was determined whether the sequences were conserved in the A. flavus genome, since *A. oryzae* and A. flavus are closely related [56], [57]. After using these criteria to narrow down the results of the BLAST search, six sequences were determined to be the most favorable.

**Figure 1:**
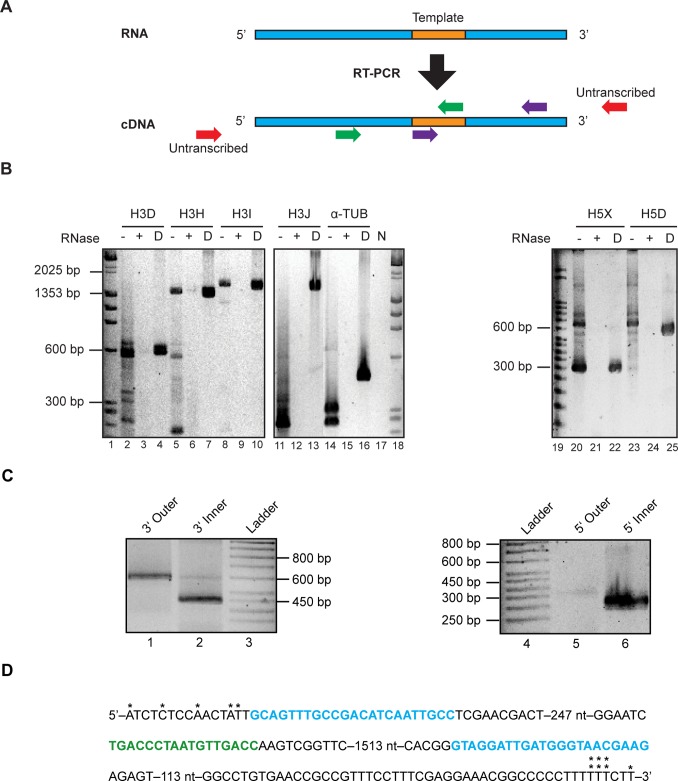
A single transcribed sequence with a putative telomerase RNA template is identified in *A. oryzae*. A. RNA was reverse transcribed using one forward primer to amplify from the template towards the 5′ end (green). Different reverse primers were used to determine roughly where the transcript ended (green indicates RT-PCR product was present, red indicates RT-PCR product does not extend that far). Likewise, a reverse primer was used to amplify from the template to the 3′ end (purple). B. Sets of three products were analyzed by gel electrophoresis: RT-PCR reactions without RNase (−), reactions with RNase (+), and PCR reactions where genomic DNA was used instead of RNA (D). The set of three reactions is labeled by the genomic sequence (e.g. “H”), the strand orientation of the first primer (e.g. “3″) and the designation of the opposite primer of the pair (e.g. “J”). Alpha-tubulin primers on either side of an intron were used as a control, where an excised intron results in smaller products in the (−) lane than in the (D) lane. C. RLM-RACE results for the 3′ end (lanes 1 and 2) and the 5′ end (lanes 5 and 6). D. The sequence in green is the anticipated template. The sequences in blue were the primers used for the RT-PCR reaction that yielded products. Asterisks indicate ends that were determined by sequence analysis, some of which were redundant at the 3′ end.

We then determined whether any of the six candidate TER template sequences were transcribed. Two sets of primers were designed for RT-PCR: one set amplified a fragment ∼200 nt upstream of the template sequence and the other set amplified a fragment ∼200 nt downstream of the template (Figure 1A). One of the candidate sequences (termed ‘H’) was indeed transcribed, as evident by RT-PCR results (Figure 1B). When additional primers were used to amplify the TER sequence 3′ to the template (H3D, H3H, H3I, and H3J) a product of predicted size 1567 bp was obtained (Figure 1B, lane 8), but not a product of 1775 bp (Figure 1B, lane 11). Therefore, the 3′ end of the transcript was between 1567 and 1775 bp downstream of the template. Likewise, when the 5′ end was similarly examined, a product of predicted size 302 bp was obtained (Figure 1B, lane 20), whereas not a product of 580 bp (Figure 1B, lane 23). Together these results indicate the length of the putative TER transcript is between 1869 and 2355 nt. Since no transcripts were detected by RT-PCR corresponding to any of the other candidate sequences (data not shown), we determined that this was the putative telomerase RNA in *A. oryzae*.

### The *Aspergillus oryzae* TER is among the Longest Mapped to Date

Having identified a putative telomerase RNA, we used RNA Ligase Mediated Rapid Amplification of cDNA Ends (RLM-RACE) to more accurately map the 5′ and 3′ ends of the *A. oryzae* RNA (Figure 1C). For the 5′-RACE a primer was ligated onto the 5′ end of isolated RNA. For the 3′-RACE, a product was obtained only upon polyadenylation of the 3′ end by E. coli poly(A) polymerase. This result is consistent with all or most transcripts lacking poly A in vivo, as found for yeast TERs [58], [59]. To increase the specificity and amount of PCR products, we used nested primers in a second PCR reaction to amplify the products of the first PCR reaction. For the 3′ end, a product was obtained near 600 bp for the outer reaction (Figure 1C, lane 1) and near 450 bp for the inner reaction (Figure 1C, lane 2), consistent with a difference between the two primers of 151 bp. For the 5′ end, a faint product is seen above 350 bp for the outer reaction and an inner nested reaction provided an intense product below the 350 bp marker. The distance between the outer and inner primers is 59 bp. The PCR products were cloned, and 5 clones from the 5′ end and 7 clones from the 3′ end were sequenced, revealing some variability at the 5′ and 3′ ends (within 14 nucleotides at the 5′ end, and within 6 nucleotides at the 3′ end) (Figure 1D). This range of heterogeneity has been seen in other TERs [17], [60].

From these sequences we can deduce that the longest *A. oryzae* TER transcript is 2035 nt, consistent with the RT-PCR results and about the size of the longer Candida TERs [16], [24]. BLAST analysis indicated that the entire 2035 nt coding sequence of TER fits into an intergenic region of the genome that lacks significant open reading frames (ORFs), approximately 580 bp upstream of a zinc finger protein, Zpr1, and approximately 400 bp downstream of a TATA box interacting protein. In addition, once the 2035 nt TER was folded using the RNA folding algorithm Mfold [50], all but 2 nts of the anticipated 17 nt template were present in a predicted single-stranded region, consistent with a sequence used as a template for telomeric repeat synthesis.

### Identifying TER Sequences in Other Filamentous Fungi

We used the BLAST utility to perform a sequence search of the genomes of other filamentous fungi using the TER sequence. We identified sequences with a significant degree of sequence similarity to that of TER in 12 filamentous fungi, comprising Aspergillus aculeatus, A. carbonarius, A. clavatus, N. fischeri, A. flavus, A. fumigatus, A. kawachii, A. nidulans, A. niger, A. sojae, A. terreus, and Penecillium chrysogenum (this collective group of organisms here will be referred to as the ‘Aspergilli’). Importantly, all these candidate TER orthologs contained a putative template sequence. In addition, examination of these sequences revealed that in nearly all cases, these sequences reside in intergenic regions (except for P. chrysogenum, where two ORFs were present within the predicted TER sequence, coding for hypothetical proteins with no identified conserved domains. A TER sequence overlapping with an ORF was found in *Arabidopsis thaliana* [20]).

**Figure 2:**
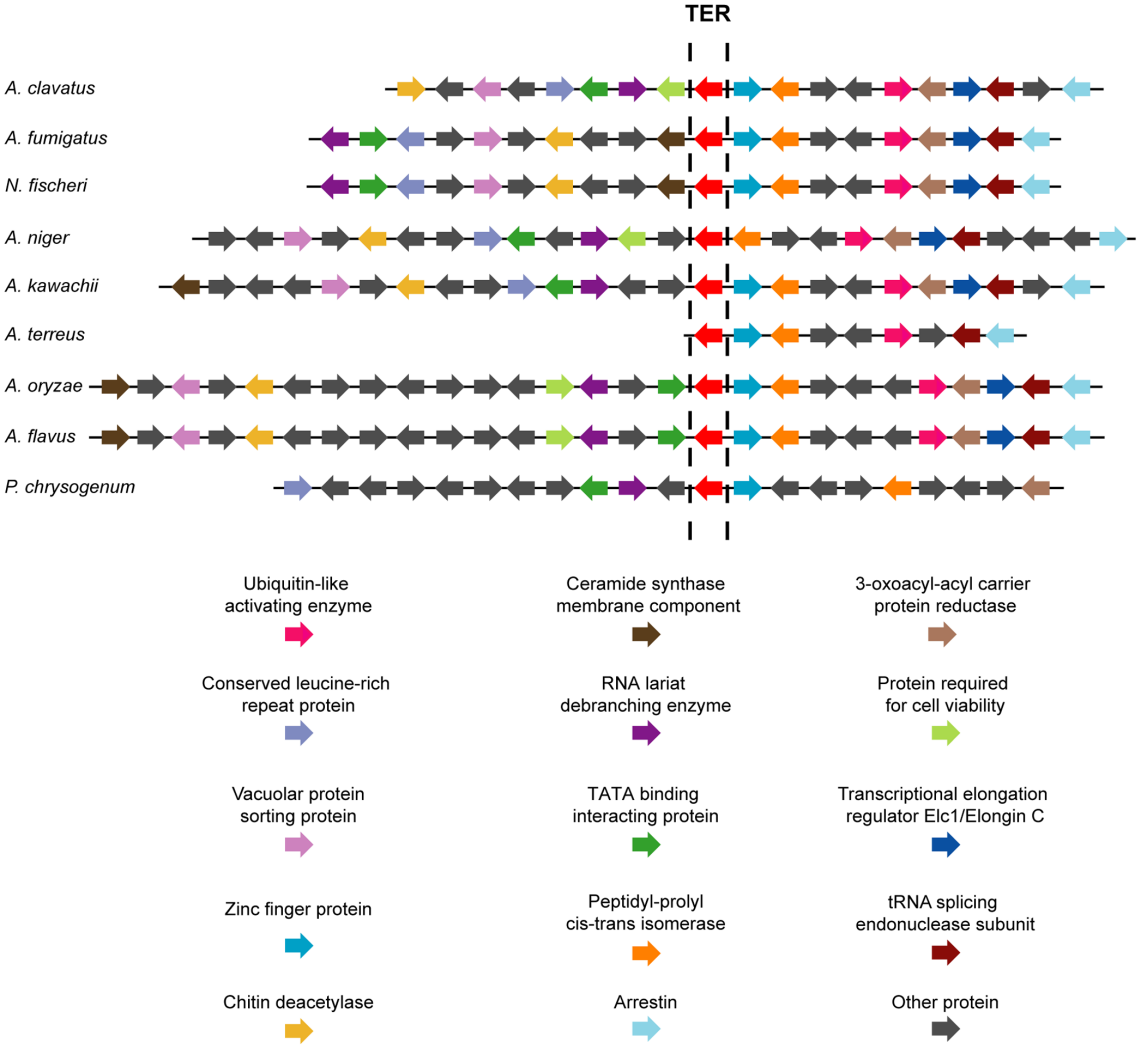
Strongly syntenous regions surround nine of the filamentous fungi TERs. The region to the right of TER shows more conservation of genes than the region to the left of TER. Chromosome VIII of A. nidulans contains TER, but no synteny is exhibited with these nine fungi, although five of the syntenous proteins were found dispersed across chromosome VIII of A. nidulans: vacuolar protein sorting protein, 3-oxoacyl-acyl carrier protein reductase, peptidyl-prolyl cis-trans isomerase, arrestin.

Strong synteny was observed in the chromosomal regions around the putative TER genes in nine of ten of the fungal genomes, providing more evidence in support of the identified sequences being orthologs (Figure 2). The synteny map also revealed deletions, insertions, or inversions of genomic fragments that include one or several genes, demonstrating the dynamics of the genome during evolution of these fungi. Interestingly, *A. nidulans* did not share synteny with the other *Aspergillius* spp. genomes. However, since there was sufficient sequence conservation with the *A. oryzae* sequence, the A. nidulans TER was easily identified.

### Aspergilli TERs Contain Short Conserved Sequences Important for TER Biogenesis

We searched outside of the TER sequences to identify conserved sequences that might play a regulatory role. Alignment of the thirteen *Aspergillus* spp. TER sequences revealed two conserved sequences upstream of the 5′ mapped end of the *A. oryzae* TER that might function as promoters (Figure S1). However, promoter sequences are poorly defined in *Aspergillus* spp [46] and the rest of the filamentous fungi, and thus remain speculative.

**Figure 3:**
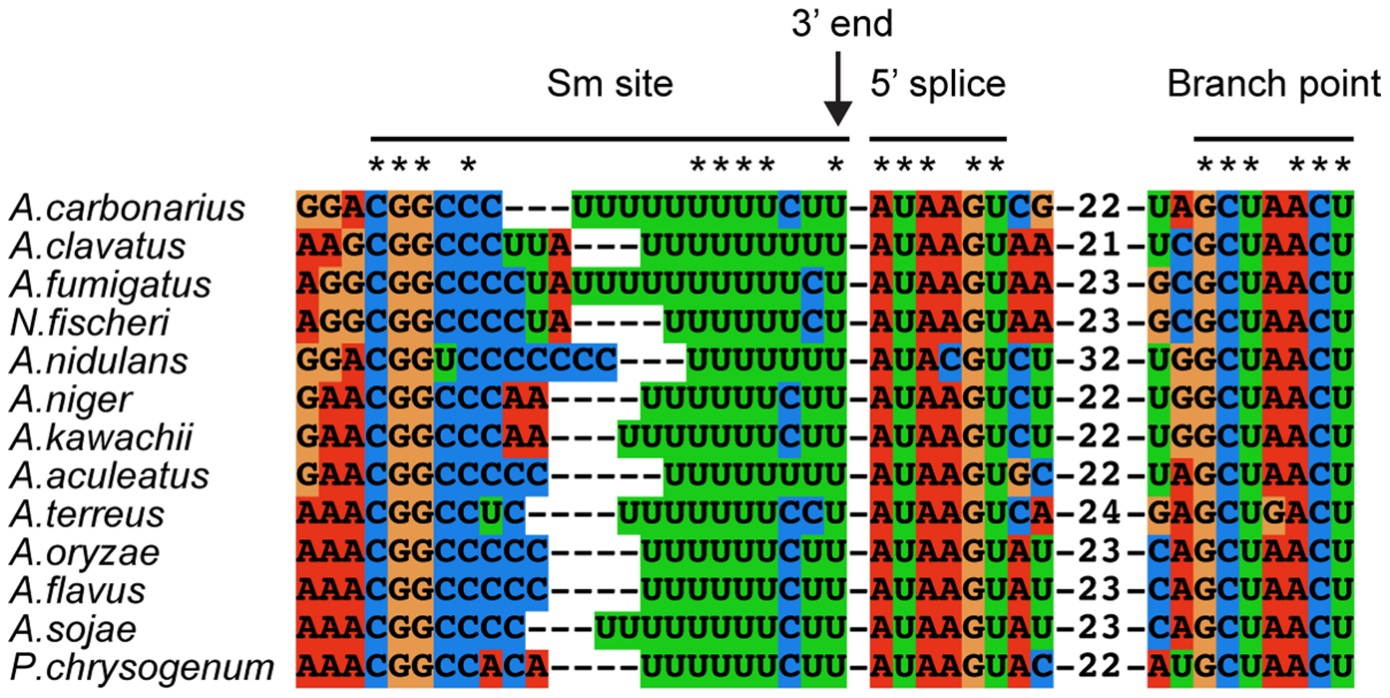
The 3′ end of the Aspergilli TERs contain conserved elements that function in TER processing. The Sm site is slightly variable, while the 5′ splice site and branch point are much more conserved within the Aspergilli. Numbers within the sequence indicate the linker nucleotides between the 5′ splice site and the branch point. Asterisks indicate conserved nucleotides in all 13 species.

We searched for sequences near the 3′ end of the *Aspergillus* spp. TERs that might be involved in Sm binding. In yeast, such a single-stranded site has been found to be important for 3′ end processing and stabilization of yeast TERs [58], [61], [62]. The yeast Sm site consensus sequence AU_5–6_GR [63] is reflected in nearly all yeast TERs: AAU_5_GG in *Saccharomyces* [60], [61], AACCAU_5–6_GG in *Kluyveromyces* [64], AU_6_GG in *Schizosaccharomyces pombe* [17], and GAU_3–4_G in *Candida* [16]. A similar sequence, CGGC_3–5_U_7–12_, is conserved at the 3′ ends of the Aspergilli TERs (Figure 3). Through the use of RNAalifold [49] and Mfold [50] this anticipated Sm site was predicted to be single stranded. The Aspergillus Sm site contains a longer tract of uridines than the yeast sequences with some adenosine and cytosine residues. However, it should be noted that the budding yeast Sm site is flexible and able to accommodate mutations while keeping its sequence functionality [63].

Adjacent to the Sm site we identified a conserved 5′ splice site AUAAGU (AUACGU in A. nidulans) and further downstream a conserved splicing branch point sequence GCUAACU (GCUGACU in A. terreus), as found in *S. pombe* and *Candida* TERs [16], [58] (Figure 3). The presence of these two sequence elements suggests that the 3′ end of the *Aspergillus* spp. TERs are processed along a similar pathway to that of S. pombe TER, using an incomplete splicing reaction [16], [58], [62].

Based on the mapped ends of the *A. oryzae* TER, the conservation of sequences close to the 5′ and 3′ ends, and the alignment of TER sequences, we predicted the ends of the other 12 TERs. We estimated the lengths of these TERs between about 1930 nt to 2130 nt, with the exception of A. nidulans, which was predicted to be about 1590 nt. This is corroborated by Qi et al. who experimentally determined the sequence to be 1584 nt [21]. Having defined the ends of the sequences, we performed a pairwise alignment of each of the TER sequences with the *A. oryzae* TER sequence, revealing between 51.5% to 99.5% sequence identity.

### The Aspergilli Utilize a Vertebrate Core Sequence to Synthesize TTAGGG-containing Telomeric Repeats

**Figure 4:**
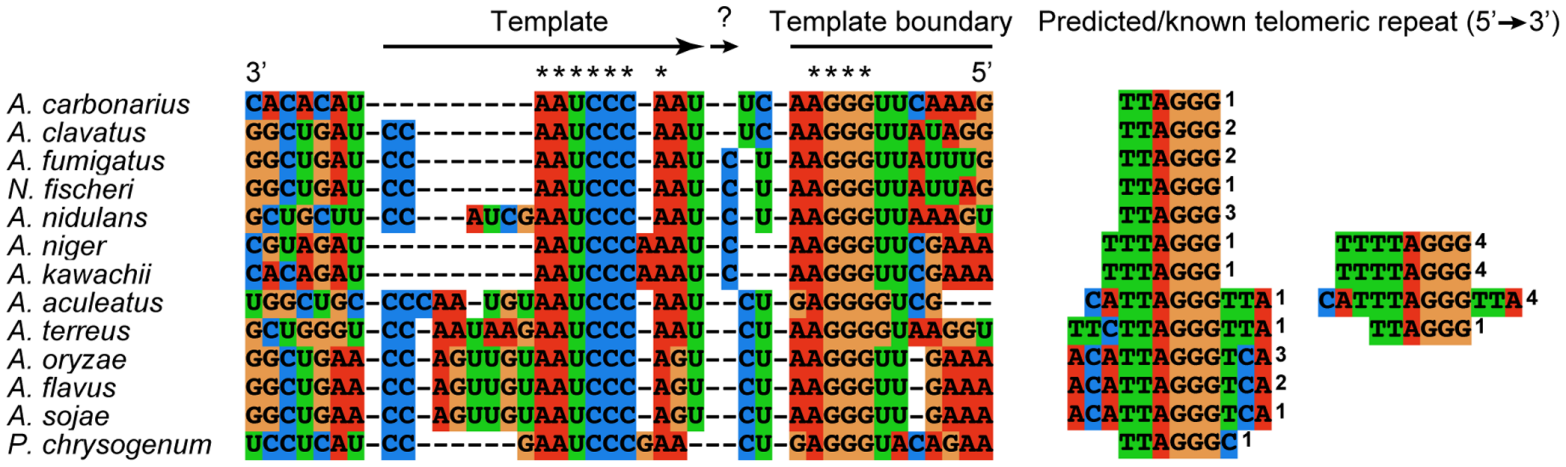
Templates and template boundaries are conserved in the Aspergilli TERs. The TER template must contain a short repeated sequence at its 5′ and 3′ ends for alignment with the telomere end. This information allowed us to identify or confirm what telomeric repeats the template sequences of these organisms could synthesize, depending on whether the telomeric repeat sequence was already identified. For example, in *A. carbonarius* the template sequence begins with UAA at the 3′ end and ends with UAA at the 5′ end of the template, which would synthesize TTAGGG. It is unknown whether A. fumigatus, N. fischeri, A. nidulans, A. niger, and A. kawachii incorporate the C before the template boundary into their templates. Superscripts to the right of the telomeric sequences indicate the manner in which the telomeric repeats were determined or predicted: ^1^Proposed by our lab based on template sequence; ^2^Proposed by researchers based on telomeric sequence (*A. clavatus* from the fungal genome database at Broad Institute, http://www.broadinstitute.org/science/data#, [56], *A. fumigatus* [40], and *A. flavus* [65]); ^3^Identified by Bal31digestion and southern blot (*A. nidulans* [22], *A. oryzae* [23]); ^4^Proposed by our lab based on genome sequence data.

The presence of telomeric repeats in the sequenced genomes of several *Aspergillus* spp. species, including A. clavatus [56], A. flavus [65], A. fumigatus [40], A. nidulans [22], and *A. oryzae* [23], allowed us to accurately predict the precise sequence used by telomerase as a template for telomere synthesis. Using ClustalX we aligned the TER sequences and determined the region that would be used as a template based on the known telomeric repeats. We also determined the direct repeats on the boundaries of the template, 3–6 nt long in the Aspergilli TERs, used for alignment of all TERs to the telomere (Figure 4; Figure S2). Interestingly, the alignment of the template sequences revealed the presence of a conserved portion of the vertebrate template, 5′-CCCUAA-3′, in all 13 species. In A. carbonarius, A. clavatus, A. fumigatus, N. fischeri, and A. nidulans the template is predicted to direct the synthesis of an identical telomeric repeat to the vertebrate repeat, 5′-TTAGGG-3′, while A. fumigatus and N. fischeri even contain the exact template sequence of human TER, 5′-CUAACCCUAAC-3′. A. aculeatus and A. terreus TERs can potentially synthesize the vertebrate telomeric repeats or longer repeats: 5′-CATTAGGGTTA-3′ and 5′-TTCTTAGGGTTA-3′, respectively.

We searched the A. aculeatus genome for telomeric sequences that had not yet been identified in order to determine whether the telomeres contained 5′-TTAGGG-3′ or 5′-CATTAGGGTTA-3′. The *A. aculeatus* pre-publication genome sequence (Joint Genome Institute) appears as though the longer telomeric repeat is utilized rather than the shorter sequence. In fact, the telomeric repeats of *A. aculeatus* appear consistently to incorporate an extra T nucleotide, 5′-CATTTAGGGTTA-3′ (Figure 4). We also searched the *A. terreus* genome, but we did not find sequences resembling telomeres within the sequenced genome, thus we were unable to determine whether the shorter or longer sequence is utilized. Telomeric sequences were also found for *A. kawachii* [44] and a different strain of *A. niger* (ATCC 1015, Joint Genome Institute) [66]. Similar to *A. aculeatus*, the *A. kawachii* and *A. niger* sometimes incorporate an extra T to their telomeric repeats predicted based on the template sequences. This finding suggests slippage of telomerase after copying the last two A nucleotides of the template or misalignment while initiating a new telomeric repeat. Such imperfect pairing at the 3′ end of the template may be stabilized by additional base pairing between the TER sequence downstream of the template and the telomeric repeat, as shown in *K. lactis* [67].

The overall conservation of a core vertebrate telomeric repeat in all 13 species suggests that it provides essential telomeric functions shared with vertebrates by virtue of specific interactions with telomeric proteins or specific structures, such as G-quadruplexes [68]. This is in contrast to the highly divergent yeast telomeric sequences, showing essentially no conservation with the vertebrate sequence [15].

### Aspergilli TERs have a Yeast-like Template Boundary Element

A template boundary element (TBE) defines the 5′ end of the telomerase template where termination occurs, and thus dictates the DNA sequence incorporated onto telomeres [28]. In yeast, the TBE consists of a stable helix nearly adjacent to the template. In vertebrates, the TBE is generally located a few nucleotides upstream of the template, consisting of two separate elements: one upstream of the template and one downstream of the pseudoknot domain [26]. In the Aspergilli TERs, we identified a yeast-type TBE – a stable helix supported by phylogenetic covariations and located mostly two nucleotides from the 5′ end of the template (Figure 4; Figure S2). The loop connecting the two strands of the helix is shorter than those present in yeast and in one case, A. aculeatus, the template boundary forms a very short stem loop, similar in size to that formed in ciliate TERs [27]. The apical end of the arm emerging from the template boundary is known to bind the Ku protein in *Saccharomyces* sensu stricto and *Candida glabrata* [24], [69] or to contain another functional element termed Reg2 in *Kluyveromyces* [70], yet the arm of the Aspergillus TERs appears to be rather short, lacking any of these elements.

### The Aspergilli TER Pseudoknot Contains U-A·U Base Triples as Found in Yeast and Vertebrate TERs

**Figure 5:**
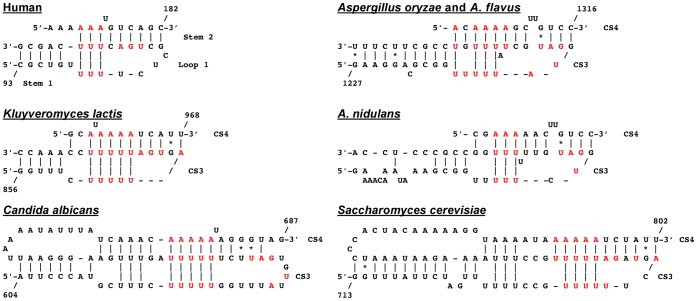
Important pseudoknots are conserved in the filamentous fungi. *A. oryzae/A. flavus* and *A. nidulans* are shown in comparison to yeast and human sequences**.** Red nucleotides indicate U-A·U base triples in addition to nucleotides fitting the conserved proposed pseudoknot consensus [16]. Numbered nucleotides for the *A. oryzae/A. flavus* pseudoknot are specific to *A. oryzae*. See Figure S3 for additional Aspergilli pseudoknots.

A pseudoknot with U-A·U base triples is a conserved feature of yeast and vertebrate TERs, and is also predicted to form in ciliate TERs [29], [30], [31], [32]. The pseudoknot has been shown to interact with TERT and is believed to aid in positioning of the telomeric substrate and the template at the catalytic site [30], [32], [71]. In Aspergilli TERs, we identified conserved sequences that can potentially form a stable pseudoknot structure (Figures 5, S1, and S3). These sequences have been termed as CS3 and CS4 due to the similarity they exhibit with the CS3 and CS4 of yeast TERs [16], [72]. CS3 contains stretches of successive uracils while the CS4 contains a stretch of adenines, which potentially form 3–4 U-A·U base triples (Figure 5, S1, and S3). In addition, a C-G·U base triple can potentially form [29]. The pseudoknot is smaller in size than those found for the Candida and Saccharomyces TERs, yet larger than the pseudoknots of vertebrate species [14], [16], [73]. Within the *Aspergillus* spp. CS3 loop there is some conservation of the consensus sequence (AKUN_0–2_GAU; K = U or G) [16], and particularly the GAU element. The pseudoknots from the *Aspergillus* spp. TERs provide more information regarding the sequence and structure conservation found within all TERs, highlighting the importance of the pseudoknot in telomerase activity.

### A Compact Aspergilli Three-way Junction Mimics the Vertebrate CR4/CR5 Domain

**Figure 6:**
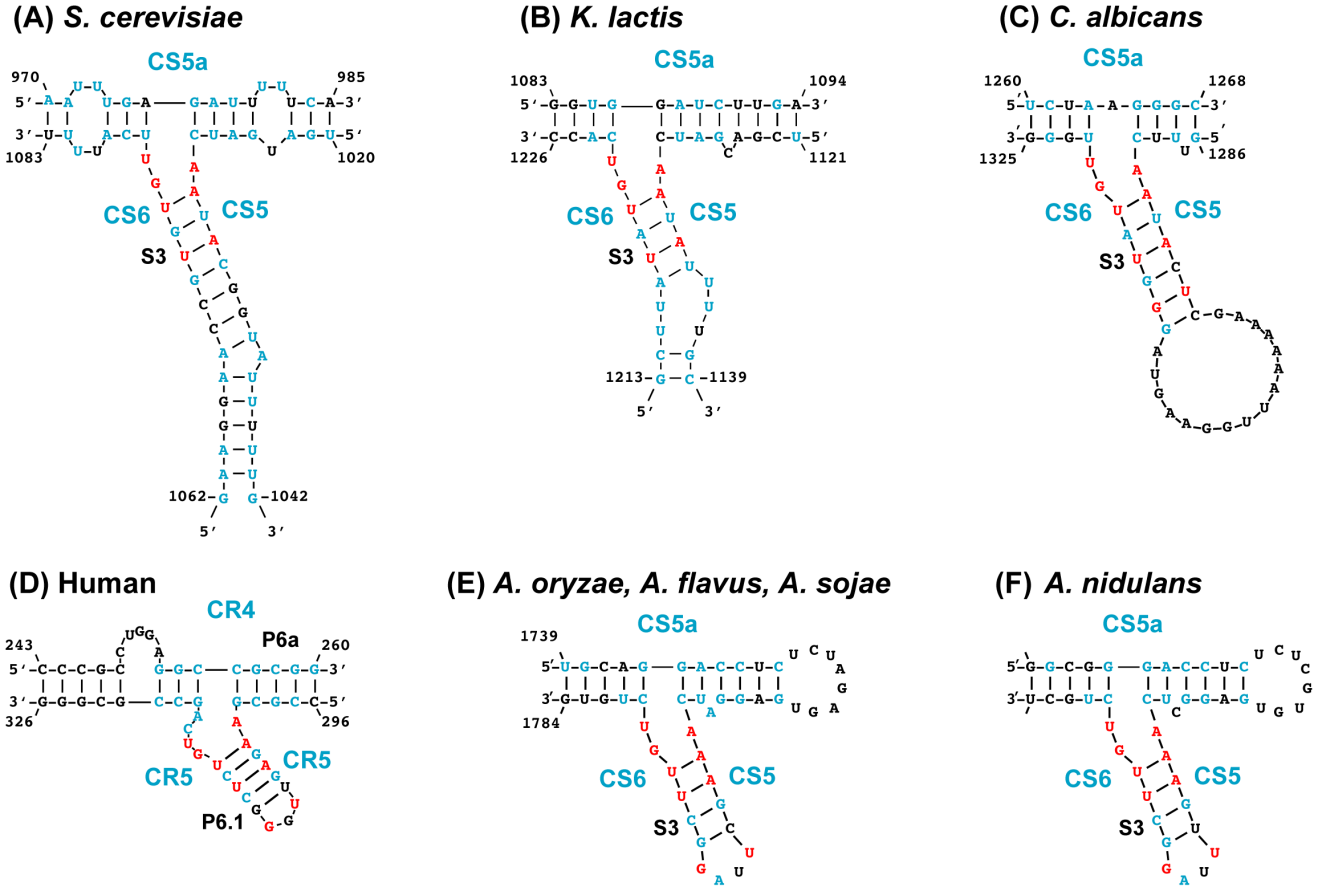
Conservation of the three-way junction. *A. oryzae*/*A. flavus*/*A. soaje* and *A. nidulans* are shown in comparison to yeast and human sequences. For the yeast and human sequences the same coloring scheme is used from Gunisova et al. [16]. For the Aspergilli TERs nucleotides in blue are conserved for 12 of 13 Aspergilli. Combining information from the TERs examined by Gunisova et al. [16], the nucleotides in red are conserved in 63 of 68 TERs examined. Numbered nucleotides for the *A. oryzae/A. flavus/A. sojae* three-way junction are specific to *A. oryzae*. See Figure S4 for additional Aspergilli three-way junctions.

Covariation-based secondary structure prediction by RNAalifold [49] revealed a three-way junction (TWJ) element in all *Aspergillus* spp. TERs formed by conserved sequences identified as CS5 and CS6, due to their similarity in yeast TERs [16], [34] (Figure 6 and S4). Interestingly, the TWJ observed in *Aspergillus* spp. is much more compact than the yeast TWJs and similar in size to the vertebrate CR4/CR5 domain. Particularly stem 3 is similar to the vertebrate p6.1 [16], [33], [34]. Furthermore, nine nucleotides are conserved in the same positions in stem 3/p6.1 and the junction across the Aspergilli and vertebrates (eight in A. terreus). Stem p6.1 has been found important for interaction with the telomerase RNA binding domain of the TERT [74]. These results demonstrate the importance of the precise TWJ structure and suggest a conservation of the TWJ function between filamentous fungi and vertebrates.

### Conclusions

**Figure 7:**
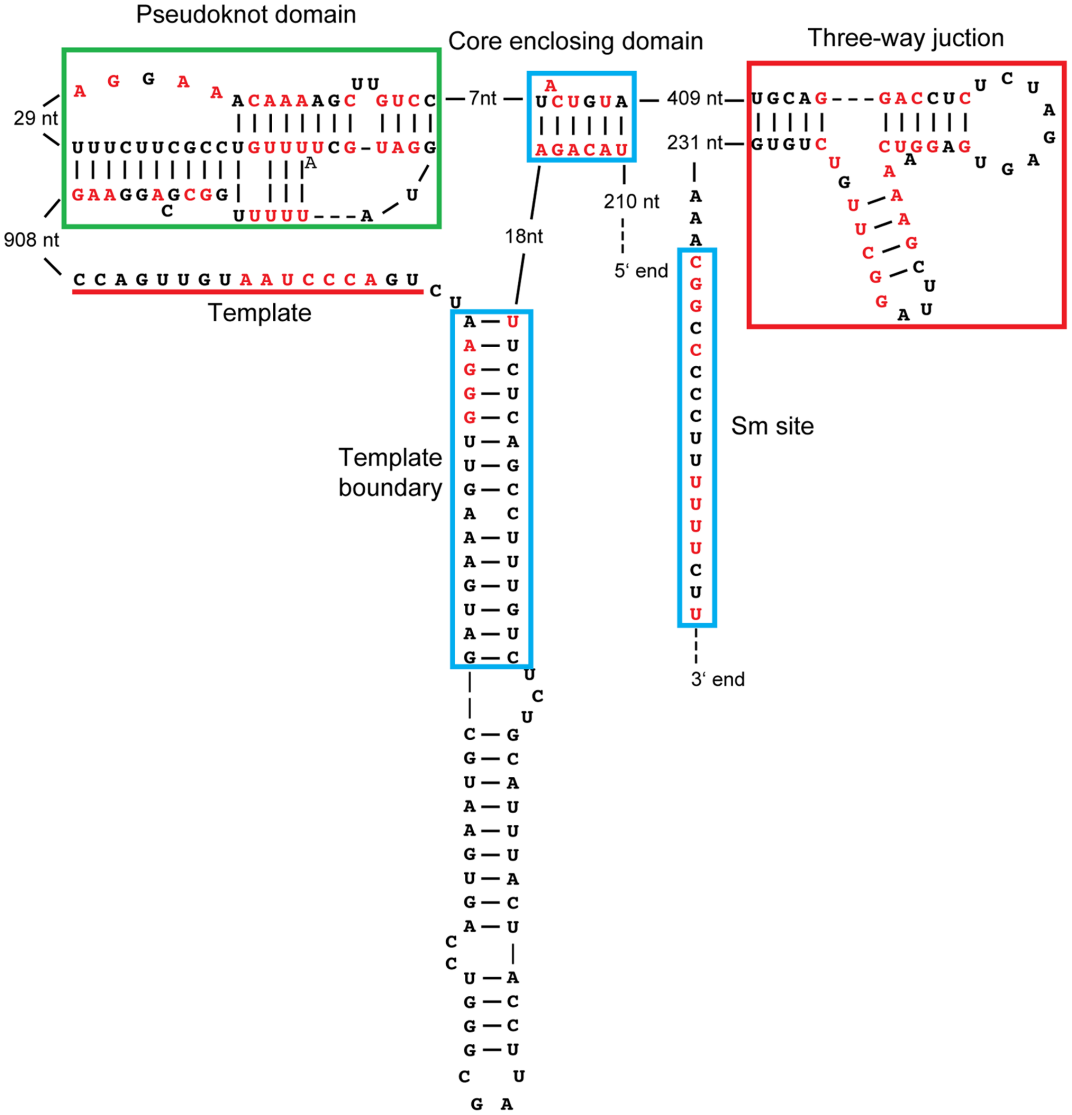
Phylogenetic-based secondary structure prediction revealed organization and functional elements conserved with other TER sequences. Boxed regions indicate similarity of the *A. oryzae* TER to other TERs: blue-yeast; red-vertebrates, green-similarity to both yeast and vertebrate. Red nucleotides indicate conservation across all 13 of the Aspergillus TERs.

In this study we report the TER sequences from a group of filamentous fungi and demonstrate how these sequences have features in common with vertebrates as well as yeasts. These TERs are similar in length to the Candida TERs, being among the longest known in any organism. Covariation-based secondary structure prediction reveals features resembling TERs from the yeasts: the template boundary, Sm site, 5′ splice site and branch point (Figure 7, blue). Interestingly, several other features are closer to vertebrate TERs: stem 3/p6.1 of the TWJ and the telomeric sequence TTAGGG copied by the TER template (Figure 7, red). The pseudoknot shares similarity with both yeast and vertebrates (Figure 7, green).

Interestingly, an evolutionary relationship may be indicated when the telomeric proteins are compared between humans, yeasts, and the Aspergilli. Humans use TRF1 and TRF2 to bind double stranded telomeric DNA, whereas budding yeast use Rap1 and fission yeast use Taz1 [75]. Budding and fission yeast both encode another protein, Tbf1, which binds double stranded DNA containing the sequence TTAGGG and located mostly in subtelomeric regions [76], [77]. When the telomeres of *S. cerevisiae* were altered to contain TTAGGG repeats instead of the typical redundant yeast repeats, the protein Tbf1, instead of Rap1, was bound to the telomere [78]. Examining the Aspergilli genome with BLAST for the human TRF proteins and the fission yeast Taz1 only revealed the *Aspergillus* Tbf1 proteins. When examining the Aspergilli genomes for the budding yeast Rap1, the *Aspergillus* Rap1 proteins had the strongest similarities though they appear to conserve only one DNA binding site while the budding yeast Rap1 has two DNA binding sites [75]. This suggests that the Aspergilli telomeres bind Tbf1 since their telomeres contain a core sequence of TTAGGG. Taken together, these observations support the notion that the vertebrate repeat represents the primordial telomere, while the deviation of the yeast telomere sequence is linked to the evolution of Rap1 as a direct telomere binding protein [79]. Since Aspergilli telomeres and telomerase have common features with humans, understanding telomere biology in filamentous fungi may elucidate common aspects in human cells.

## Supporting Information

Figure S1 **Clustal X alignment of 10 Aspergilli TERs.** Conserved functional sequences are outlined in blue. Strongly conserved sequences without a determined function are outlined in red.
(PDF)

Figure S2 **Folded template boundary and templates of Aspergilli TERs.** In nearly all cases the template boundary seems to consist of 8 base pairs. For all sequences the longest possible template is underlined. The conserved vertebrate telomeric core, 5′-CCCUAA-3′, is present in red.
(AI) Click here for additional data file.

Figure S3 **Pseudoknots of Aspergilli TERs.** These are the pseudoknots not shown in Figure 5. The same coloring scheme is followed as in Figure 5.
(PDF)

Figure S4 **Three-way junctions of Aspergilli TERs.** These are the three-way junctions not shown in Figure 6. The same coloring scheme is followed as in Figure 6.
(AI) Click here for additional data file.

Table S1 Primers used in this study on *A. oryzae*.
(PDF)
