# Supplementary material for: Identification of Telomerase RNAs from Filamentous Fungi Reveals Conservation with Vertebrates and Yeasts
Source: PLoS One. 2013 Mar 14;8(3):e58661. doi: 10.1371/journal.pone.0058661 (PMC3603654; doi:10.1371/journal.pone.0058661)
Supplement: Figure S1 — Clustal X alignment of 10 Aspergilli TERs. Conserved functional sequences are outlined in blue. Strongly conserved sequences without a determined function are outlined in red. (PDF) [file pone.0058661.s001.pdf]

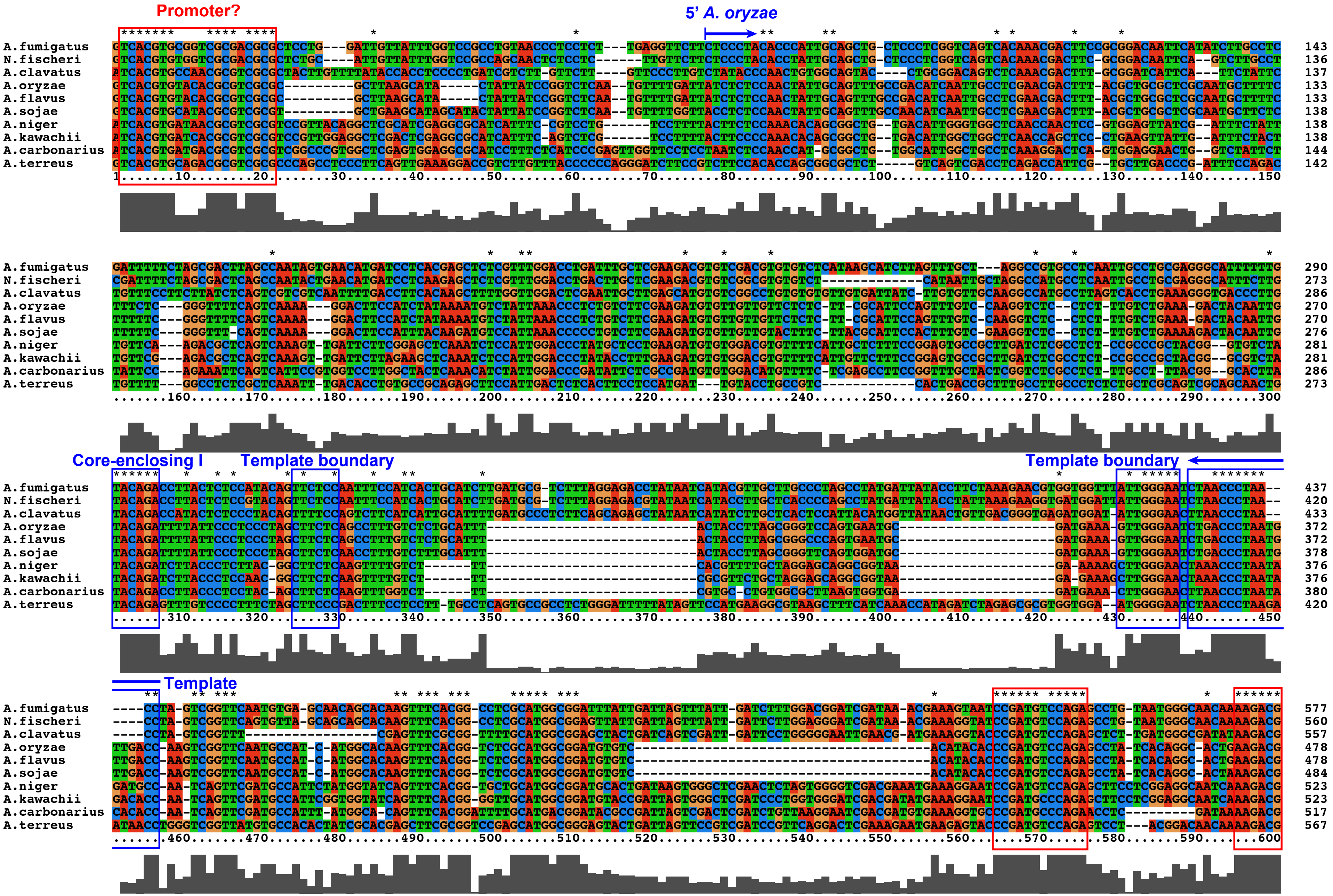
Promoter?
5' A. oryzae
Core-enclosing I
Template boundary
Template boundary
Template
A.fumigatus
N.fischeri
A.clavatus
A.oryzae
A.flavus
A.sojae
A.niger
A.kawachii
A.carbonarius
A.terreus

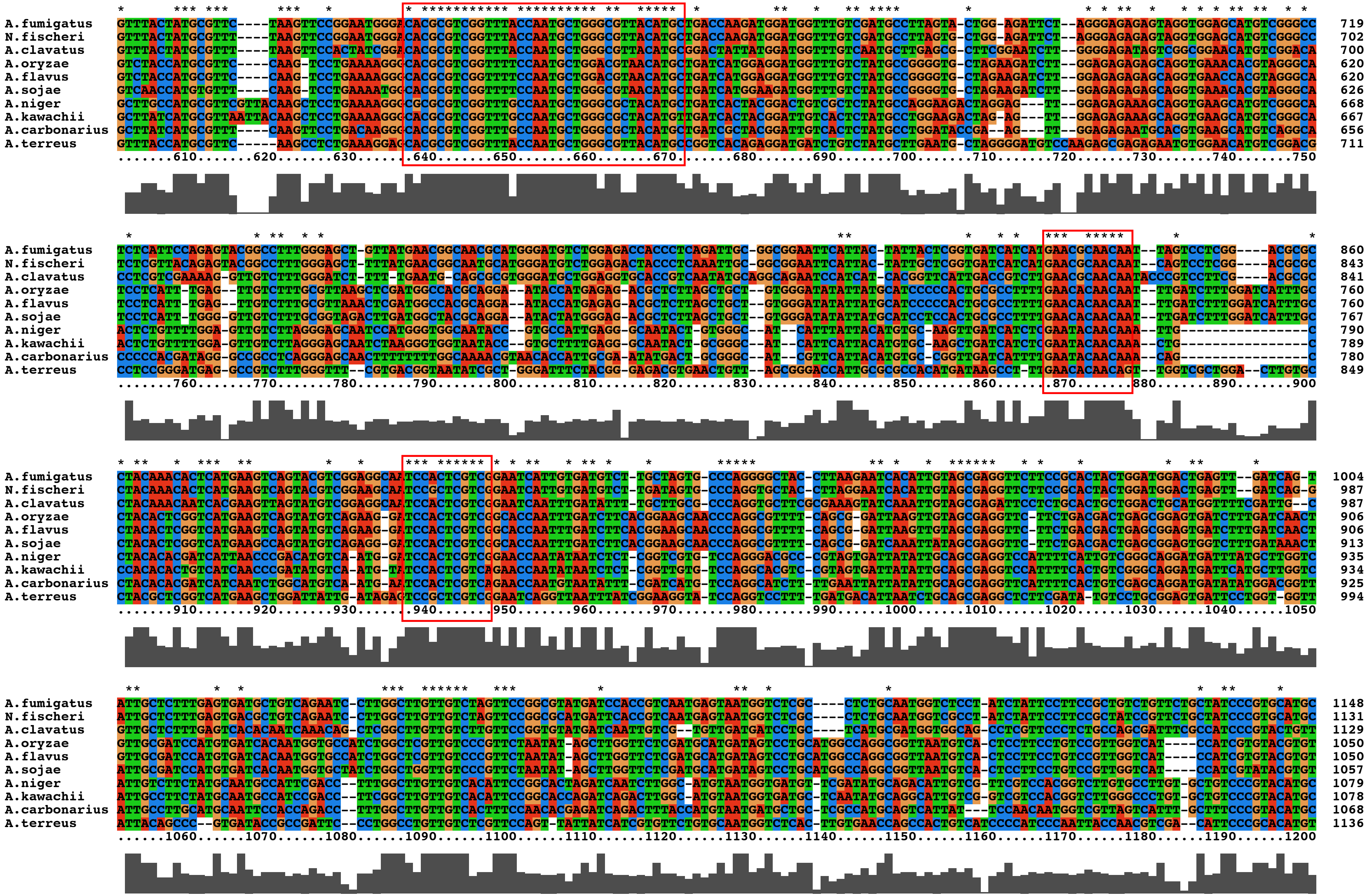
A.fumigatus 719
N.fischeri 702
A.clavatus 700
A.oryzae 620
A.flavus 620
A.sojae 626
A.niger 668
A.kawachii 667
A.carbonarius 656
A.terreus 711
A.fumigatus 860
N.fischeri 843
A.clavatus 841
A.oryzae 760
A.flavus 760
A.sojae 767
A.niger 790
A.kawachii 789
A.carbonarius 780
A.terreus 849
A.fumigatus 1004
N.fischeri 987
A.clavatus 987
A.oryzae 906
A.flavus 906
A.sojae 913
A.niger 935
A.kawachii 934
A.carbonarius 925
A.terreus 994
A.fumigatus 1148
N.fischeri 1131
A.clavatus 1129
A.oryzae 1050
A.flavus 1050
A.sojae 1057
A.niger 1079
A.kawachii 1078
A.carbonarius 1068
A.terreus 1136

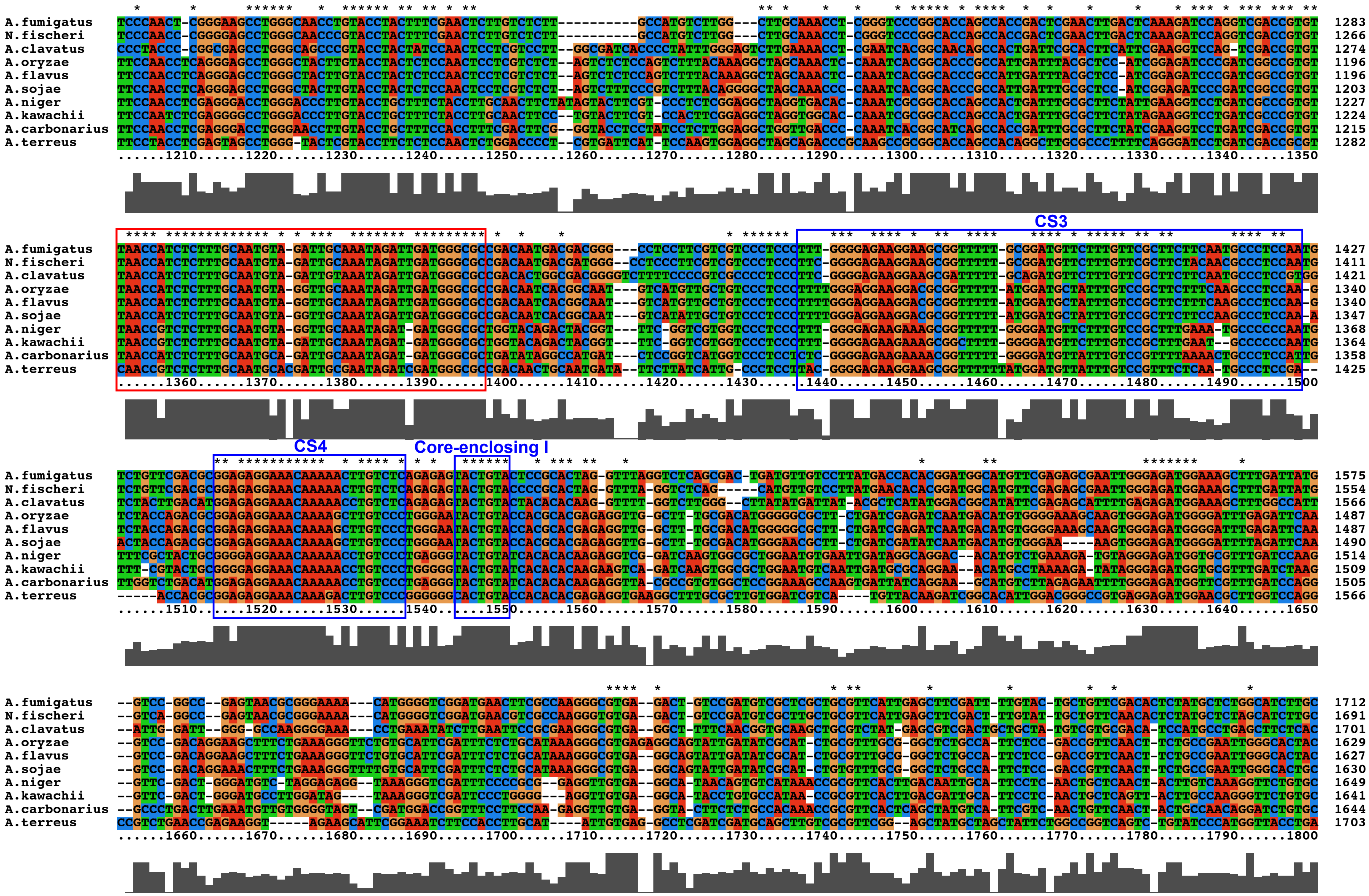
A.fumigatus
N.fischeri
A.clavatus
A.oryzae
A.flavus
A.sojae
A.niger
A.kawachii
A.carbonarius
A.terreus
CS3
CS4
Core-enclosing I

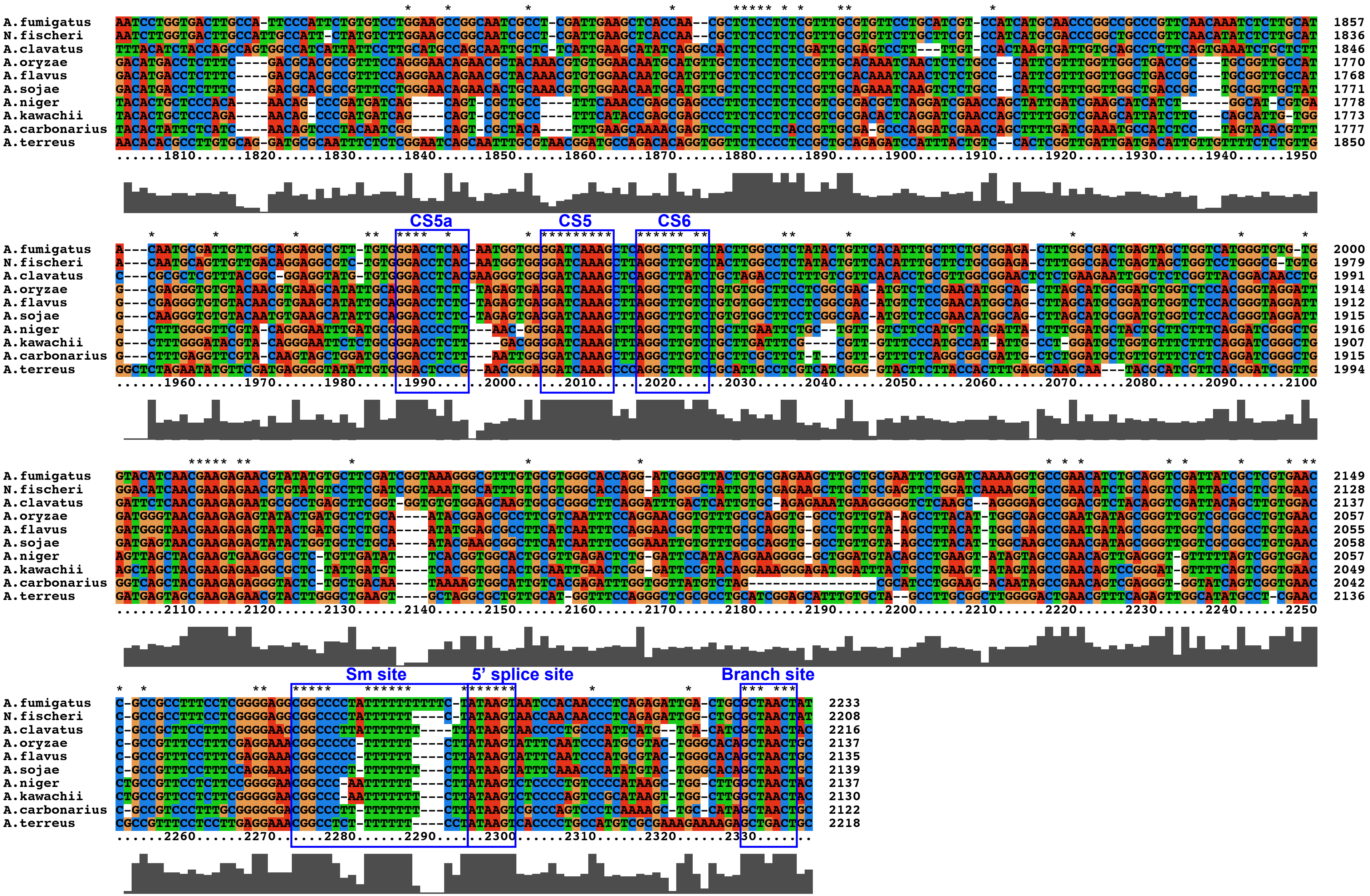
A.fumigatus
N.fischeri
A.clavatus
A.oryzae
A.flavus
A.sojae
A.niger
A.kawachii
A.carbonarius
A.terreus
CS5a
CS5
CS6
Sm site
5' splice site
Branch site
1857
1836
1846
1770
1768
1771
1778
1773
1777
1850
2000
1979
1991
1914
1912
1915
1916
1907
1915
1994
2149
2128
2137
2057
2055
2058
2057
2049
2042
2136
2233
2208
2216
2137
2135
2139
2137
2130
2122
2218
