# Supplementary material for: Identification of Telomerase RNAs from Filamentous Fungi Reveals Conservation with Vertebrates and Yeasts
Source: PLoS One. 2013 Mar 14;8(3):e58661. doi: 10.1371/journal.pone.0058661 (PMC3603654; doi:10.1371/journal.pone.0058661)
Supplement: Figure S3 — Pseudoknots of Aspergilli TERs. These are the pseudoknots not shown in Figure 5. The same coloring scheme is followed as in Figure 5. (PDF) [file pone.0058661.s003.pdf]

A. fumigatus

```
                                     UU
                   5'- A C A A A A A C  G U C U-3' CS4
                       | | | | | | | |  * | | *
3'-C U U C U U C G C U U G U U U U U G  U A G G
   | | | | * | | | | * | | | | |C           /
5'-G A A G G A G C G G | | | | |           C   CS3
              A        U U U U U - - -G  -
```

N. fischeri

```
                                     UU
                   5'- A C A A A A A C  G U C U-3' CS4
                       | | | | | | | |  * | | *
3'-C A U C U U C G C U U G U U U U U G  U A G G
   |   | | * | | | | * | | | | |C           /
5'-G A A G G A G C G G | | | | |           C   CS3
              A        U U U U U - - -G  -
```

A. clavatus

```
                                     UU
                   5'- A C A A A A A C  G U C U-3' CS4
                       | | | | | | | |  * | | |
3'-C U U C U U C G C U U G U U U U U G  U A G A
   | | | | * | | | | | | | | | |C           /
5'-G A A G G A G C G A | | | | |           C   CS3
              A        U U U U U - - -G  -
```

A. sojae

```
                                     UU
                   5'- A C A A A A G C  G U C C-3' CS4
                       | | | | | | | |  * | | |
3'-C U U C U U C G C C U G U U U U C G  U A G G
   | | | | * | | | | | | | | | |A           /
5'-G A A G G A G C G G | | | | |           U   CS3
              C        U U U U U - - -A  -
```

Penicillium chryosogenum

```
                                     CU
                   5'- A C A A A A A C  G U C C-3' CS4
                       | | | | | | | |  * | | |
3'-A C A C U U C G C G U G U U U U U G  U A G G
         | * | | | |   | | | | |G           /
5'-G A A G G A G C G A | | | | |           C   CS3
              A        U U U U U - - -C  -
```

A. niger

```
                                     CU
                   5'- A C A A A A A C  G U C C-3' CS4
                       | | | | | | | |  * | | |
3'-A A G U U U C G C C U G U U U U U G  U A G G
         * | | | | | | | | | | |C           /
5'-G A A G A A G C G G | | | | |           G   CS3
              A        U U U U U - - -G  -
```

A. kawachii

```
                                     CU
                   5'- A C A A A A A C  G U C C-3' CS4
                       | | | | | | | |  * | | |
3'-A A G U U U C G C C U G U U U U U G  U A G G
         * | | | | | |   | | | |C           /
5'-G A A G A A G C G G C | | | |           G   CS3
              A          U U U U - - -G  -
```

A. carbonarius

```
                                     CU
                   5'- A C A A A A A C  G U C C-3' CS4
                       | | | | | | | |  * | | |
3'-A A A U U U U G C C U G U U U U U G  U A G G
         * | | | | | | | | | | |A           /
5'-G A A G A A A C G G | | | | |           G   CS3
              A        U U U U U - - -G  -
```

A. terreus

```
                                     UU
                   5'- A C A A A G A C  G U C C-3' CS4
                       | | | | | | | |  * | | |
3'-A C U C U U U G C C U G U U U U U G  U A G G
       | | * | * | | | | | | | |A           /
5'-G A A G G A G C G G | | | | |           U   CS3
              A        U U U U U - U -A  -
```

A. aculeatus

```
                             A       UA
                   5'- A U C A A A A C  G U C C-3' CS4
                       |   | | | | | |  * | | |
3'-C U U U G C C U G U U U G U U U U G  U A G G
   | | | *     | | | * |   | | |            /
5'-G A A G G A G C G G |   | | |           U   CS3
              A        U U U U U - - -A  -
```
