# Supplementary material for: Identification of Telomerase RNAs from Filamentous Fungi Reveals Conservation with Vertebrates and Yeasts
Source: PLoS One. 2013 Mar 14;8(3):e58661. doi: 10.1371/journal.pone.0058661 (PMC3603654; doi:10.1371/journal.pone.0058661)
Supplement: Table S1 — Primers used in this study on A. oryzae. (PDF) [file pone.0058661.s005.pdf]

Table S1. Primers used in this study on *A. oryzae*

A. RT-PCR Experiments			
Candidate Sequence	Side of template	Direction	Sequence (5'→ 3')
A	5'-1	Frwd	GTAATGCCGAGCACCACGATTG
		Rev	TAATGTTGACCCTAAATGTGCAAG
	3'-1	Frwd	TTAGGGTCAACATTACTGCTACTC
		Rev	CTATCTTGGAGGGCATGACCG
	5'-2	Frwd	GGGACAATCTACATCCATGAGG
		Rev	TAATGTTGACCCTAAATGTGCAAG
	3'-2	Frwd	TAGGGTCAACATTACTGCTACTC
		Rev	CTAGCATTGTCGCAGCTGCAC
	5'-3	Frwd	TGGCTTTTGCACCTCGACTCG
		Rev	CCACGGATACTGGGGAAAGG
	3'-3	Frwd	GGTGACGTTTTGATTGGATCCC
		Rev	CTGGCAGTGTCTAAAAGTCTCC
B	5'	Frwd	TCAGTACACAGGATTTTCTCCTG
		Rev	TAATGTTGACCCTAAAGAAAATCTC
	3'	Frwd	CTTTAGGGTCAACATTATCACTTG
		Rev	CAGAGACGCACGAAGCATCG
D	5'-1	Frwd	CTTTGAAGATGATTACCCCTTGG
		Rev	AGGGTCAACATTAAGGGTGTTACC
	3'-1	Frwd	ACCCTAATGTTGACCCTCATGC
		Rev	CGATCGTAAGAAGCCGGTGAAC
	5'-2	Frwd	CGTAACAGGATCTGGACATGC
		Rev	ACCCTAATGTTGACCCTCATGC
	3'-2	Frwd	AGGGTCAACATTAGGGTGTTAC
		Rev	GGACAGATACTCGCACATTTGG
	5'-3	Frwd	GGCCTGATCTAGCATAAAAGCG
		Rev	GTATTCGCAAGTGCAAGAGATC
	3'-3	Frwd	CTCCGAACAGAGGTTAGTTG
		Rev	CACAGAAGACGGTCGCGACG
E	5'	Frwd	CTACGATAGGCGGCAATGGG
		Rev	TAATGTTGACCCTAATGAAGTGG
	3'	Frwd	CATTAGGGTCAACATTAAAAGGTG
		Rev	GACTTGTGAAACCGGTTGGGG
G	5'	Frwd	GGCCATCTTCAAGAGTAACCAG
		Rev	CATTAGGGTCAACATTCCTGTC
	3'	Frwd	AATGTTGACCCTAATGCCAATAC
		Rev	GGGATGAGATACTAACTGGAAC
H	5'	A-Frwd	CCAAGGTCTCCTCTTTGTCTG
		X-Frwd	CAGTTTGCCGACATCAATTGCC
		D-Frwd	AGTAGTGGAAACTTGCGTGTGC
		F-Frwd	CCAAAGGATAATGCCGTGAAGC
		Rev	GGTCAACATTAGGGTCAGATTC
	3'	Frwd	TGACCCTAATGTTGACCAAGTC
		A-Rev	GACCGTCTTCAGTGCCTGTG
		B-Rev	CAAACCATCCTCCATGATCAGC

Table S1 (*continued*)

H	3'	C-Rev	GACCGAGTGTAGGCAAATGATC
		D-Rev	GACAACGAGCCAGATGGCAC
		E-Rev	CCGTGATTTGGAGTTTGCTAGC
		F-Rev	GAGGGCTTGAAAGAAGCGGAC
		G-Rev	CGCCCTTTATGCAGAGAAATCG
		H-Rev	GAATGGGCAGAGAGTTGATTTG
		I-Rev	CTTCGTTACCCATCAATCCTAC
		J-Rev	CAGTTAGCTGTGCCCAGTACG
		K-Rev	GTCGCATGGAGTCGAACAGTC
		L-Rev	GAGGTCTATAACGCGCAAATGG
α-Tubulin control	n/a	Frwd	GTTGCCAGATCGCCAATTCTTG
		Rev	TGTTCGAGGCATCCTCCTTGC

B. 5' RLM-RACE

**Name**	**Sequence (5'→ 3')**
Outer	GGTCAACATTAGGGTCAGATTCCC
Inner	GGACCCGCTAAGGTAGTAAATGC

C. 3' RACE

**Name**	**Sequence (5'→ 3')**
Outer	CGTGAGAGGCAGTATTGATATCGC
Inner	CATGTTGCTCTCCTCTCCGTTGC
